# From LaH_10_ to room–temperature superconductors

**DOI:** 10.1038/s41598-020-58065-9

**Published:** 2020-01-31

**Authors:** M. Kostrzewa, K. M. Szczęśniak, A. P. Durajski, R. Szczęśniak

**Affiliations:** 1 Institute of Physics, Jan Długosz University in Częstochowa, Ave. Armii Krajowej 13/15, 42-200 Częstochowa, Poland; 2 Faculty of Chemistry, University of Warsaw, Pasteura 1, 02-093 Warsaw, Poland; 3 Institute of Physics, Częstochowa University of Technology, Ave. Armii Krajowej 19, 42-200 Częstochowa, Poland

**Keywords:** Condensed-matter physics, Superconducting properties and materials

## Abstract

Thermodynamic parameters of the LaH_10_ superconductor were an object of our interest. LaH_10_ is characterised by the highest experimentally observed value of the critical temperature: ${{\boldsymbol{T}}}_{{\boldsymbol{C}}}^{{\boldsymbol{a}}}={\bf{215}}$ K (*p*_*a*_ = 150 GPa) and ${{\boldsymbol{T}}}_{{\boldsymbol{C}}}^{b}={\bf{260}}$ K (*p*_*b*_ = 190 GPa). It belongs to the group of superconductors with a strong electron-phonon coupling (*λ*_*a*_ ~ 2.2 and *λ*_*b*_ ~ 2.8). We calculated the thermodynamic parameters of this superconductor and found that the values of the order parameter, the thermodynamic critical field, and the specific heat differ significantly from the values predicted by the conventional BCS theory. Due to the specific structure of the Eliashberg function for the hydrogenated compounds, the qualitative analysis suggests that the superconductors of the La_*δ*_X_1−*δ*_H_10_-type (LaXH-type) structure, where X ∈ {Sc, Y}, would exhibit significantly higher critical temperature than *T*_*C*_ obtained for LaH_10_. In the case of LaScH we came to the following assessments: ${{\boldsymbol{T}}}_{{\boldsymbol{C}}}^{{\boldsymbol{a}}}\in \left\langle {\bf{220}},{\bf{267}}\right\rangle$ K and ${{\boldsymbol{T}}}_{{\boldsymbol{C}}}^{{\boldsymbol{b}}}\in \left\langle {\bf{263}},{\bf{294}}\right\rangle$ K, while the results for LaYH were: ${{\boldsymbol{T}}}_{{\boldsymbol{C}}}^{{\boldsymbol{a}}}\in \left\langle {\bf{218}},{\bf{247}}\right\rangle$ K and ${{\boldsymbol{T}}}_{{\boldsymbol{C}}}^{{\boldsymbol{b}}}\in \left\langle {\bf{261}},{\bf{274}}\right\rangle$ K.

## Introduction

The experimental discovery of the high-temperature superconducting state in the compressed hydrogen and sulfur systems H_2_S (*T*_*C*_ = 150 K for *p* = 150 GPa) and H_3_S (*T*_*C*_ = 203 K for *p* = 150 GPa)^1,2^ accounts for carrying out investigations, which can potentially lead to the discovery of a material showing the superconducting properties at room temperature. For the first time, the possibility of the existence of the superconducting state in hydrogenated compounds was pointed out by Ashcroft in 2004^3^. It was stated in his second fundamental work concerning the high-temperature superconductivity, following his first work written in 1968, in which he propounded the existence of the high-temperature superconducting state in metallic hydrogen^4^. The superconducting state in hydrogenated compounds is induced by the conventional electron-phonon interaction. This fact made possible the theoretical description of the superconducting phase in H_2_S and H_3_S even prior to carrying out the suitable experiments^5,6^. The detailed discussion with respect to the thermodynamic properties of the superconducting state occurring in H_2_*S* and H_3_*S* one can find in references^7–17^.

In 2018, there were held the groundbreaking experiments, which confirmed the existence of the superconducting state of extremely high values of the critical temperature in the LaH_10_ compound: ${T}_{C}^{a}=215$ K for *p*_*a*_ = 150 GPa and ${T}_{C}^{b}=260$ K for *p*_*b*_ ∈ (180–200) GPa (and then ${T}_{C}^{c} \sim 250$ K for *p*_*c*_ ~ 170 GPa^18^). It was proved on the theoretical basis^19^ that the results achieved by Drozdov *et al*.^20^ can be related to the induction of the superconducting phase in the $R\bar{3}m$ structure (*T*_*C*_ = 206–223 K). The experimental results reported by Somayazulu *et al*.^21^ should be related to the superconducting state induced in the $Fm\bar{3}m$ structure, where the critical temperature can potentially reach even the value of 280 K. From the materials science perspective, the achieved results imply that all possible actions should be taken in order to examine the hydrogen-containing materials with respect to the existence of the high-temperature superconducting state at room temperature. Attention should be paid to the importance of the discovery of the high-temperature superconducting state in LaH_10_ because La can form stable hydrogenated compounds with other metals. Such materials can exhibit so large hydrogen concentration, that they are presently taken into account as basic components of the hydrogen cells intended for vehicle drives^22^.

The purpose of this work is, firstly, to present the performed analysis of the thermodynamic properties of the superconducting state in the LaH_10_ compound. We took advantage of the phenomenological version of the Eliashberg equations, for which we fitted the value of the electron-phonon coupling constant on the basis of the experimentally found *T*_*C*_ value. Our next step consisted in examining the hydrogenated compounds of the La_*δ*_*X*_1−*δ*_*H*_10_-type (LaXH-type) on the basis of the achieved results in order to find a system with an even higher value of the critical temperature. Taking into account the structure of the Eliashberg function for hydrogenated compounds, with its distinctly separated parts coming from the heavy elements and from hydrogen, we assumed X to be Sc or Y, what would, in our opinion, fill the gap in the Eliashberg function occurring within the range from about 40 meV to 100 meV. A significant increase in the value of critical temperature should take place as a consequence.

## Computational Details, Results and Discussion

The thermodynamic parameters of the LaH_10_ superconductor were calculated by means of Eliashberg equations on the imaginary axis^23^:

1 $${\Delta }_{n}{Z}_{n}=\pi {k}_{B}T{\sum }_{m=-M}^{M}\frac{[K\left({\Omega }_{n}-{\Omega }_{m}\right)-{\mu }^{\star }\left({\Omega }_{m}\right)]}{\sqrt{{\Omega }_{m}^{2}+{\Delta }_{m}^{2}}}{\Delta }_{m},$$

and

2 $${Z}_{n}=1+\pi {k}_{B}T{\sum }_{m=-M}^{M}\frac{K\left({\Omega }_{n}-{\Omega }_{m}\right)}{\sqrt{{\Omega }_{m}^{2}+{\Delta }_{m}^{2}}}\frac{{\Omega }_{m}}{{\Omega }_{n}}{Z}_{m}.$$

**Figure 1:**
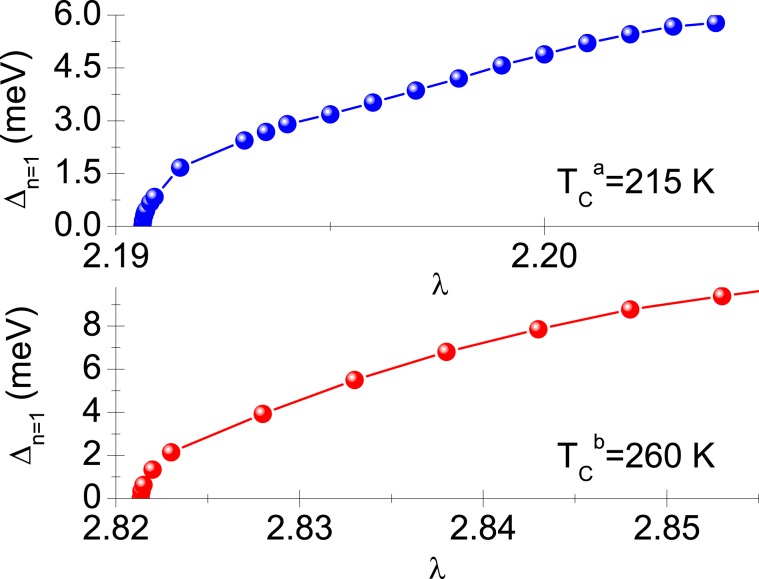
The dependence of the maximum value of the order parameter on the electron-phonon coupling constant. We consider two cases: ${T}_{C}^{a}=215$ K (*p*_*a*_ = 150 GPa) and ${T}_{C}^{b}=260$ K (*p*_*b*_ = 190 GPa).

The symbols ${\Delta }_{n}=\Delta \left(i{\Omega }_{n}\right)$ and ${Z}_{n}=Z\left(i{\Omega }_{n}\right)$ denote the order parameter and the wave function renormalization factor, respectively. The quantity Ω_*n*_ represents the Matsubara frequency: ${\Omega }_{n}=\pi {k}_{B}T\left(2n-1\right)$, where *k*_*B*_ is the Boltzmann constant. The pairing kernel is defined by: $K\left({\Omega }_{n}-{\Omega }_{m}\right)=\lambda \frac{{\Omega }_{C}^{2}}{{\left({\Omega }_{n}-{\Omega }_{m}\right)}^{2}+{\Omega }_{C}^{2}}$, where *λ* denotes the electron–phonon coupling constant. We determined the value of *λ* on the basis of experimental data^20,21^ and the condition: ${\left[{\Delta }_{n=1}\right]}_{T={T}_{C}}=0$. The fitting between the theory and the experimental results is presented in Fig. 1. We obtained *λ*_*a*_ = 2.187 for *p*_*a*_ = 150 GPa and *λ*_*b*_ = 2.818 for *p*_*b*_ = 190 GPa. The symbol Ω_*C*_ represents the characteristic phonon frequency, its value being assumed as Ω_*C*_ = 100 meV.

The repulsion between electrons is modeled by the function: ${\mu }^{\star }\left({\Omega }_{m}\right)={\mu }^{\star }\theta \left({\Omega }_{C}-| {\Omega }_{m}| \right)$, where ${\mu }^{\star }$is the Coulomb pseudopotential (${\mu }^{\star }$ = 0.1). The quantity Ω_*C*_ denotes the cut–off frequency (Ω_*C*_ = 1 eV). The Eliashberg equations were solved for the Matsubara frequency equal to 1000. We used numerical methods presented in the previous paper^24^. In the considered case, we obtained stable equation solutions for *T* ≥ *T*_0_ = 15 K.

**Figure 2:**
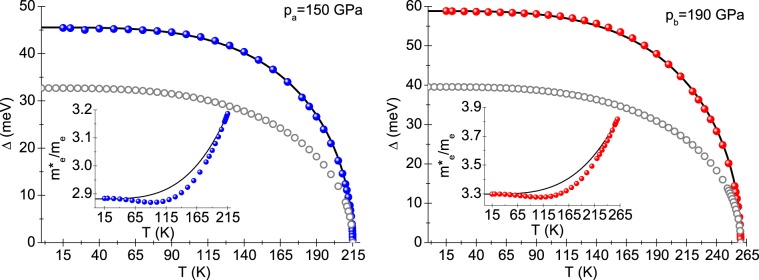
The dependence of the order parameter on temperature. The insets present the influence of temperature on the value of effective electron mass to the band electron mass ratio. Blue or red disks represent numerical results. Black curves were obtained from the analytical formulae: $\Delta \left(T\right)=\Delta \left({T}_{0}\right)\sqrt{1-{\left(T/{T}_{C}\right)}^{\Gamma }}$ and ${m}_{e}^{\star }/{m}_{e}=\left[Z\left({T}_{C}\right)-Z\left({T}_{0}\right)\right]{\left(T/{T}_{C}\right)}^{\Gamma }+Z\left({T}_{0}\right)$, where $Z\left({T}_{C}\right)=1+\lambda$, *Γ*_*a*_ = 3.5 and *Γ*_*b*_ = 3.4. The predictions of the BCS theory we marked with grey circles.

Figure 2 illustrates the full dependence of the order parameter on temperature. Physical values of the order parameter were calculated from the equation: $\Delta \left(T\right)=Re\left[\Delta \left(\Omega =\Delta \left(T\right)\right)\right]$, while the function of the order parameter on the real axis ($\Delta \left(\Omega \right)$) was determined using the solutions of the Eliashberg equations on the imaginary axis and the analytical continuation method described in the reference^25^. It can be easily seen that the order parameter curves determined within the Eliashberg formalism differ significantly from the curves resulting from the BCS theory^26,27^. These differences arise from the very high value of the electron-phonon coupling constant of the superconductor, what is mirrored by the high value of the dimensionless ${R}_{\Delta }=2\Delta \left({T}_{0}\right)/{k}_{B}{T}_{C}$ ratio, namely ${R}_{\Delta }^{a}=4.91$ and ${R}_{\Delta }^{b}=5.25$. Let us recall that within the BCS theory we come to the result: ${\left[{R}_{\Delta }\right]}_{BCS}=3.53$, however the BCS theory approximates well the experimental results for *λ* < 0.5.

We plotted the temperature dependence of the effective electron mass (${m}_{e}^{\star }$) to the band electron mass (*m*_*e*_) ratio in the insets in Fig. 2. The value of the ${m}_{e}^{\star }/{m}_{e}$ ratio is given with good approximation by the value of 1 + *λ*^28^.

**Figure 3:**
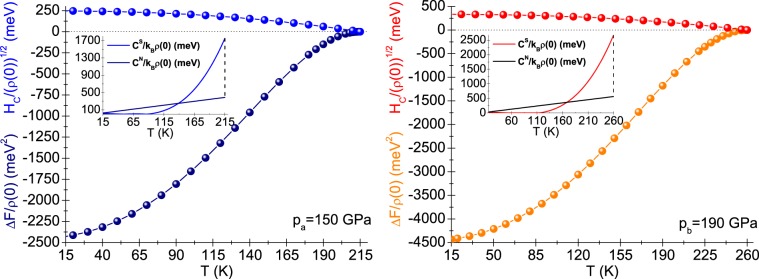
(Navy and orange line:) The difference in free energy between the superconducting and the normal state versus temperature. (Blue and red line:) Thermodynamic critical field. (Insets:) The specific heat in the superconducting and the normal states.

Figure 3 presents the results achieved for the difference in free energy between the superconducting and the normal state (*ΔF*), the thermodynamic critical field (*H*_*C*_), and the specific heat in both the superconducting (*C*^*S*^) and the normal (*C*^*N*^) states. The values of the considered quantities were calculated on the basis of formulae given in reference^28^. In particular, the difference in free energy between the superconducting and the normal state is given by:

3 $$\frac{\Delta F}{\rho \left(0\right)}=-2\pi {k}_{B}T\mathop{\sum }\limits_{n=1}^{M}(\sqrt{{\Omega }_{n}^{2}+{\Delta }_{n}^{2}}-\left|{\Omega }_{n}\right|)\times \left({Z}_{n}^{S}-{Z}_{n}^{N}\frac{\left|{\Omega }_{n}\right|}{\sqrt{{\Omega }_{n}^{2}+{\Delta }_{n}^{2}}}\right),$$

where $\rho \left(0\right)$ denotes the value of electronic density of states at Fermi surface; ${Z}_{n}^{S}$ and ${Z}_{n}^{N}$ are the wave function normalization factors for the superconducting and the normal state, respectively. Note that Δ*F* is equal to zero exactly for *T* = *T*_*C*_. This fact results from the overt dependence of free energy on solutions of Eliashberg equations (Δ_*n*_ and *Z*_*n*_) that have been adjusted to the experimental value of critical temperature by appropriate selection of electron-phonon coupling constant (see Fig. 1). Thermodynamic critical field should be calculated from the formula:

4 $$\frac{{H}_{C}}{\sqrt{\rho \left(0\right)}}=\sqrt{-8\pi \left[\Delta F/\rho \left(0\right)\right]}.$$

The difference in the specific heat between the superconducting and the normal state (Δ*C* = *C*^*S*^ − *C*^*N*^) is given by:

5 $$\frac{\Delta C\left(T\right)}{{k}_{B}\rho \left(0\right)}={-k}_{B}T\frac{{d}^{2}\left[\Delta F/\rho \left(0\right)\right]}{d{\left({k}_{B}T\right)}^{2}}.$$

The most convenient way of estimation the specific heat for the normal state is using the expression:

6 $$\frac{{C}^{N}\left(T\right)}{{k}_{B}\rho \left(0\right)}=\gamma {k}_{B}T.$$

The Sommerfeld constant take the form: $\gamma =\frac{2}{3}{\pi }^{2}\left(1+\lambda \right)$.

Deviations from the results of the BCS theory can be traced in the easiest way by determining the values of dimensionless ratios: ${R}_{H}={T}_{C}{C}^{N}\left({T}_{C}\right)/{H}_{C}^{2}\left(0\right)$ and ${R}_{C}=\Delta C\left({T}_{C}\right)/{C}^{N}\left({T}_{C}\right)$. For the LaH_10_ superconductor, we achieved the following results: ${R}_{H}^{a}=0.117$, ${R}_{H}^{b}=0.113$ and ${R}_{C}^{a}=3.51$, ${R}_{C}^{b}=3.75$. It is worth noticing that the BCS theory predicts ${\left[{R}_{H}\right]}_{BCS}=0.168$ and ${\left[{R}_{C}\right]}_{BCS}=1.43$^26–29^.

The subsequent last part of the paper discusses the question of induction of the superconducting state in a group of compounds of the La_*δ*_X_1−*δ*_H_10_–type (or LaXH–type for short). Firstly, we are going to give some criteria, which can potentially make easier the search for a material showing the required high–temperature superconducting properties. To do this, let us take into account the formula for the critical temperature valid for the BCS theory: ${k}_{B}{T}_{C}=1.13\,{\Omega }_{\max }\exp \left[-1/\rho \left(0\right)V\right]$, where ${\Omega }_{\max }$ denotes the Debye frequency and *V* stands for the pairing potential value. It can be easily noticed that the critical temperature is the higher, the greater are the values of the electron density of states at the Fermi surface, the pairing potential, and the maximum phonon frequency. Therefore it should be supposed, even at such an early stage of analysis, that special attention is to be paid to these hydrogenated compounds, for which the respective non-hydrogenated compounds (La_*δ*_X_1−*δ*_) or hydrides XH exhibit the high density of electron states at the Fermi surface. Considerations given to the pairing potential at the phenomenological level do not get us very far because this quantity is calculated in a rather complicated way, usually employing the DFT (Density Functional Theory) method.

Nevertheless, a sensible qualitative analysis can be made with respect to the influence of the atomic mass of the X element on a value of the critical temperature (since the mass of the X element determines ${\Omega }_{\max }$). In this regard, let us refer to the theoretical results obtained within the Eliashberg formalism for H_2_S and H_3_S superconductors^5,6^. They prove that contributions to the Eliashberg function (${\alpha }^{2}F\left(\Omega \right)$) coming from sulphur and from hydrogen are separated due to a huge difference between atomic masses of these two elements. To be precise, the electron-phonon interaction derived from sulphur is crucial in the frequency range from 0 meV to ${\Omega }_{\max }^{S}$ equal to about 70 meV, while the contribution derived from hydrogen (${\Omega }_{\max }^{H}=220$ meV) is significant above ~100 meV. It is noteworthy that we come upon a similar situation in the case of the LaH_10_ compound^30^. Therefore the following factorization of the Eliashberg function for the LaXH compound can be assumed:

7 $$\begin{array}{ccc}{\alpha }^{2}F\left(\Omega \right) & = & {\lambda }^{La}{\left({\frac{\Omega }{{\Omega }_{\max }}}^{La}\right)}^{2}\theta ({\Omega }_{\max }^{La}-\Omega )+{\lambda }^{X}{\left(\frac{\Omega }{{\Omega }_{\max }^{X}}\right)}^{2}\theta ({\Omega }_{\max }^{X}-\Omega )\\  &  & +{\lambda }^{H}{\left(\frac{\Omega }{{\Omega }_{\max }^{H}}\right)}^{2}\theta ({\Omega }_{\max }^{H}-\Omega ),\end{array}$$

where *λ*^La^, *λ*^X^, and *λ*^H^ are the contributions to the electron–phonon coupling constant derived from both metals (La, X) and hydrogen, respectively. Similarly, the symbols ${\Omega }_{\max }^{La}$, ${\Omega }_{\max }^{X}$, and ${\Omega }_{\max }^{H}$ represent the respective maximum phonon frequencies. The value of the critical temperature can be assessed from the generalised formula of the BCS theory^7^:

8 $${k}_{B}{T}_{C}={f}_{1}{f}_{2}\frac{{\Omega }_{ln}}{1.27}\,\exp \,\left[\frac{-1.14\left(1+\lambda \right)}{\lambda -(1+0.163\lambda ){\mu }^{\star }}\right],$$

while the symbols appearing in Eq. (8) are defined in Table 1.

**Table 1:** The quantities: *λ* (electron–phonon coupling constant), ${\Omega }_{ln}$ (logarithmic phonon frequency), Ω_2_ (second moment of the normalized weight function), *f*_1_ (strong–coupling correction function), and *f*_2_ (shape correction function) μ.

Quantity
$$\lambda =2{\int }_{0}^{+\infty }d\Omega \frac{{\alpha }^{2}\left(\Omega \right)F\left(\Omega \right)}{\Omega }$$,
$${\Omega }_{ln}=\exp \left[\frac{2}{\lambda }{\int }_{0}^{+\infty }d\Omega \frac{{\alpha }^{2}F\left(\Omega \right)}{\Omega }ln\left(\Omega \right)\right]$$,
$${\Omega }_{2}=\frac{2}{\lambda }{\int }_{0}^{+\infty }d\Omega {\alpha }^{2}F\left(\Omega \right)\Omega $$,
$${f}_{1}={\left[1+{\left(\frac{\lambda }{{\Lambda }_{1}}\right)}^{\frac{3}{2}}\right]}^{\frac{1}{3}}$$, $${f}_{2}=1+\frac{\left(\frac{\sqrt{{\Omega }_{2}}}{{\Omega }_{ln}}-1\right){\lambda }^{2}}{{\lambda }^{2}+{\Lambda }_{2}^{2}}$$,
*Λ*_1_ = 2.4 − 0.14*μ*^’^,
$${\Lambda }_{2}=\left(0.1+9{\mu }^{\star }\right)\left(\sqrt{{\Omega }_{2}}/{\Omega }_{ln}\right)$$.

Let us calculate explicitly the relevant quantities:

9 $$\lambda ={\lambda }^{La}+{\lambda }^{X}+{\lambda }^{H},$$

10 $$\begin{array}{ccl}{\Omega }_{ln} & = & \exp \left[\frac{{\lambda }^{La}}{{\lambda }^{La}+{\lambda }^{X}+{\lambda }^{H}}\left(ln({\Omega }_{\max }^{La})-\frac{1}{2}\right)\right]\times \exp \left[\frac{{\lambda }^{X}}{{\lambda }^{La}+{\lambda }^{X}+{\lambda }^{H}}\left(ln({\Omega }_{\max }^{X})-\frac{1}{2}\right)\right]\\  &  & \times \exp \left[\frac{{\lambda }^{H}}{{\lambda }^{La}+{\lambda }^{X}+{\lambda }^{H}}\left(ln({\Omega }_{\max }^{H})-\frac{1}{2}\right)\right],\end{array}$$

and

11 $${\Omega }_{2}=\frac{{\lambda }^{La}}{{\lambda }^{La}+{\lambda }^{X}+{\lambda }^{H}}\frac{{({\Omega }_{\max }^{La})}^{2}}{2}+\frac{{\lambda }^{X}}{{\lambda }^{La}+{\lambda }^{X}+{\lambda }^{H}}\frac{{({\Omega }_{\max }^{X})}^{2}}{2}+\frac{{\lambda }^{H}}{{\lambda }^{La}+{\lambda }^{X}+{\lambda }^{H}}\frac{{({\Omega }_{\max }^{H})}^{2}}{2}.$$

We are going to consider the case ${\Omega }_{\max }^{La} \sim 40\ meV < {\Omega }_{\max }^{X} < 100\ meV$. It means that we are interested in such an X element, the contribution of which to the Eliashberg function fills the gap between contributions coming from lanthanum and hydrogen. It can be assumed that 0 < *λ*^X^ < 1, while keeping in mind that *λ*^La^ = 0.68^31^. Additionally, the previous calculations discussed in the work allow to write that *λ*^La^ + *λ*^H^ is equal to *λ*_*a*_ = 2.187 for *p*_*a*_ = 150 GPa or to *λ*_*b*_ = 2.818 for *p*_*b*_ = 190 GPa. The quantity ${\mu }^{\star }$ occurring in the Eq. (8) serves now as the fitting parameter. One should remember that the formula for the critical temperature given by the Eq. (8) was derived with the use of significant simplifying assumptions (the value of the cut–off frequency is neglected, as well as the retardation effects modeled by the Matsubara frequency). Therefore the value of the Coulomb pseudopotential determined from the full Eliashberg equations usually differs from the value of ${\mu }^{\star }$ calculated analytically. The experimental data for the LaH_10_ superconductor can be reproduced using Eq. (8) and assuming that ${\mu }_{a}^{\star }=0.170$ and ${\mu }_{b}^{\star }=0.276$.

**Figure 4:**
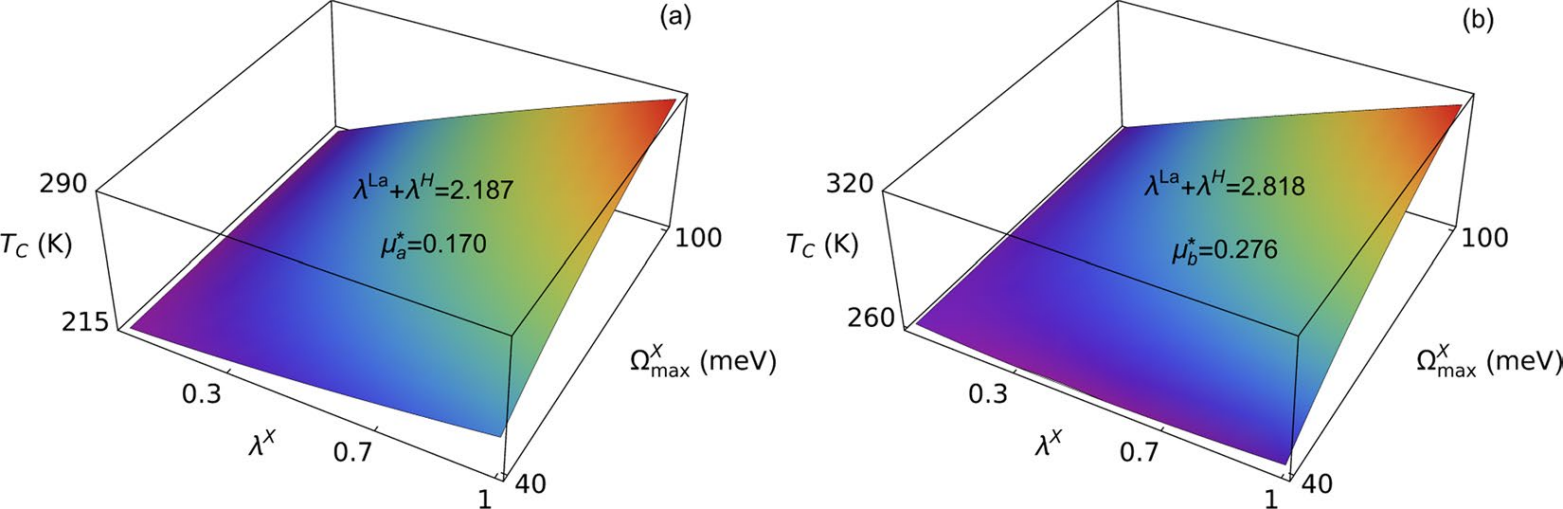
The dependence of the critical temperature on *λ*^X^ and ${\Omega }_{\max }^{X}$. Figure (**a**) presents the results for *λ*^La^+ *λ*^H^=2.187 and ${\mu }_{a}^{\star }=0.170$. Figure (**b**) is plotted for *λ*^La^+ *λ*^H^ = 2.818 and ${\mu }_{b}^{\star }=0.276$. It was assumed that ${\Omega }_{\max }^{La}=40$ meV and ${\Omega }_{\max }^{H}=290$ meV for both cases.

The achieved results are presented in Fig. 4. It is evident that taking into consideration the additional X element, which enriches the LaH_10_ composition, leads to a large increase in the critical temperature value. The estimated upper limit of the ${T}_{C}^{a}$ value is equal to 288 K for *p*_*a*_ = 150 GPa, while for *p*_*b*_ = 190 GPa we obtain ${T}_{C}^{b}=315$ K. Therefore the superconducting state can potentially exist at room temperature for both cases.

Now, let us take into account elements with the identical electron configuration at the valence shell as lanthanum, but lighter than lanthanum: scandium and yttrium, both being selected as X. Attention should be paid to the fact that the electron configuration of X, identical as in lanthanum, should minimize such changes in properties of the obtained compound which could result from changes in both the electron dispersion relation and the matrix elements of the electron-phonon interaction. Applying the formula: ${\Omega }_{\max }^{X}/{\Omega }_{\max }^{La} \sim \sqrt{{M}_{La}/{M}_{X}}$ we get ${\Omega }_{\max }^{Sc} \sim 70$ meV and ${\Omega }_{\max }^{Y} \sim 50$ meV (*M*_La_ and *M*_X_ denote atomic mass of lanthanum and the element X, i.e. Sc or Y, respectively).

**Figure 5:**
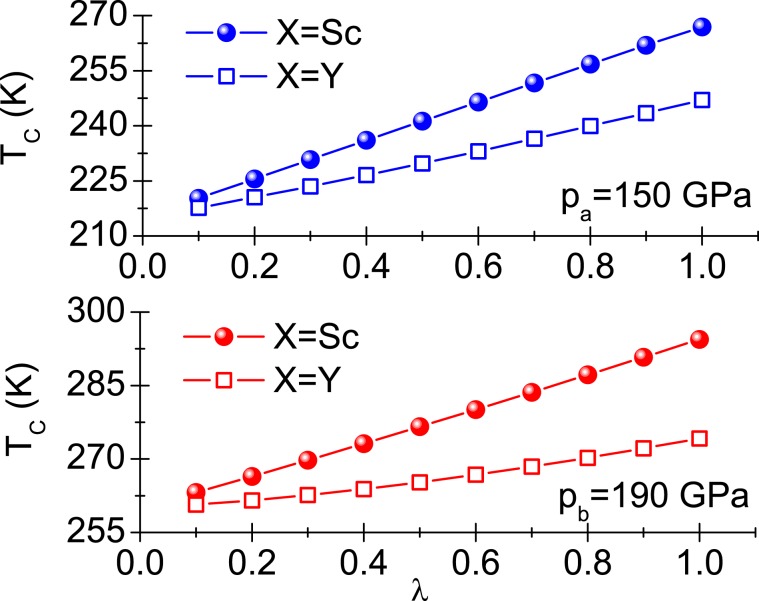
The expected range of critical temperature values for the LaScH and the LaYH compounds.

Figure 5 presents the expected range of the critical temperature values for the LaScH and the LaYH compounds. We took into account two pressure values: *p*_*a*_ = 150 GPa and *p*_*b*_ = 190 GPa. For LaScH we got: ${T}_{C}^{a}\in \left\langle 220,267\right\rangle$ K and ${T}_{C}^{b}\in \left\langle 263,294\right\rangle$ K, while the results for LaYH are as follows: ${T}_{C}^{a}\in \left\langle 218,247\right\rangle$ K and ${T}_{C}^{b}\in \left\langle 261,274\right\rangle$ K. Apparently, the significant increase in the critical temperature value should be observed in both cases. The effect of growth in the value of the critical temperature results from filling the gap in the Eliashberg function between the contributions coming from La and H, as was already stated above.

To summarize, the experimental results obtained for the LaH_10_ compound get us much closer to the purpose of obtaining the superconducting state at room temperature. The huge difference between atomic masses of lanthanum and hydrogen results in the characteristic structure of the Eliashberg function modeling the electron-phonon interaction in the considered compound, with distinctly separated parts proceeded either from lanthanum or from hydrogen. The proper selection of the additional element (X) in the LaXH compound is expected to fill the ’empty’ range of the Eliashberg function between the parts coming from La and H. In our opinion, good candidates are scandium and yttrium. These elements have the electron configuration at the valence shell exactly the same as lanthanum, and yet they are considerably lighter. Our numerical calculations suggest the possible growth in the critical temperature of the LaScH compound equal to about 52 K (150 GPa) or to about 79 K (190 GPa) as compared to the *T*_*C*_ value for the LaH_10_ compound. As far as the LaYH compound is concerned, the pertinent increase in *T*_*C*_ value can reach about 32 K for 150 GPa or about 59 K for 190 GPa.

Our results can be the starting point for the advanced DFT calculations or perhaps provide inspiration for carrying out the appropriate experimental measurements. Of course, we realize that the presented analysis is based on the semi-quantitative approximations and may raise some critical remarks. For this reason, we refer to the most significant reservations:

- *In the paper, we assume the relatively simple form of Eliashberg function, which is the linear combination of each of the contributions from La, H and X. Does this mean that any contribution related to Sc or Y will be positive?* Of course, this doesn’t have to be the case. For example, the properly selected concentration of Sc or Y atoms can lead to a decrease in the electron-phonon coupling constant. On the other hand, one should remember the results obtained for YH_10_ compound^32^,^33^. Based on the DFT method, it was found that the critical temperature for $p\in \left(250,300\right)$ GPa can exceed the room temperature (${\left[{T}_{C}\right]}_{\max } \sim 320$ K). This result suggests that a high concentration of Y atoms in the LaYH compound should not lead to a decrease in *T*_*C*_. It should also be noted that future DFT calculations (or possible experiments) should take into account different concentrations of Sc and Y atoms.
- *In the paper, we assume that the phonon frequency regime is completely decoupled (La contributes to low phonon modes and hydrogen vibration should be high). What significance will La-H, Sc-H or Y-H modes which are related to the moderate frequency regime?* In our opinion, they will contribute to the increase in the critical temperature. This will result from activating the Eliashberg function in the frequency range from ~ 40 meV to ~100 meV. This effect was clearly visible in the case of YSH_6_ and LaSH_6_ (*p* ~ 200 GPa), where the role of dopant fulfilled sulphur^34^.
- *Do the values of the Coulomb pseudopotential assumed in* Eq. (8) (${\mu }_{a}^{\star }=0.170$* and *${\mu }_{b}^{\star }=0.276$) *roughly correspond to the physical values of this parameters? In particular, are these values too low, which would lead to the significant overestimation of the critical temperature in our paper*. In this case, it is worth referring to the recently obtained DFT results for LaH_10_. In the publication^19^, the authors showed that qualitative compliance with experimental data can be obtained assuming ${\mu }^{\star }$ = 0.2 (${\left[{T}_{C}\right]}_{p=150GPa}=197$ K for the crystal structure *R*$\bar{3}$m, and ${\left[{T}_{C}\right]}_{p=200GPa}=271$ K for the crystal structure *Fm*$\bar{3}$m). In the first case, the experimental critical temperature was underestimated by 18 K (too high value of ${\mu }^{\star }$), in the second case, ${\left[{T}_{C}\right]}_{\exp }$ was revalued by 11 K (too low value of ${\mu }^{\star }$). Comparing the results obtained in the paper^19^ with ours, it is clearly seen that ${\mu }_{a}^{\star }$ and ${\mu }_{b}^{\star }$ are fairly well-obtained. In the most interesting case for LaH_10_ corresponds to the pressure of 190 GPa, taking into account the possible reduction of ${\mu }_{b}^{\star }$ suggested in^19^, the increase in the critical temperature value for the LaScH and LaYH compounds can be expected. It is worth noting that our results also correlate well with the data obtained in the paper^30^, where ${\mu }^{\star }$ = 0.22 was assumed, which allowed to reproduce the experimental critical temperature for LaH_10_ (*p* = 190 GPa).

One needs to recognise that the exact quantitative analysis of the problem discussed in our work would require to be carried out using the Eliashberg equations including the anharmonicity of the phonon system and the non-linear terms of the electron-phonon–phonon interaction, especially for such high values of the critical temperature as are observed for LaH_10_. Presently we work upon the derivation of suitable equations.
